# The effects of nano-curcumin as a nutritional strategy on clinical and inflammatory factors in children with cystic fibrosis: the study protocol for a randomized controlled trial

**DOI:** 10.1186/s13063-021-05224-6

**Published:** 2021-04-20

**Authors:** Saeedeh Talebi, Mahammad Safarian, Mahmood Reza Jaafari, Seyed Javad Sayedi, Zahra Abbasi, Golnaz Ranjbar, Hamid Reza Kianifar

**Affiliations:** 1 Department of Nutrition, Faculty of medicine, Mashhad University of Medical Sciences, Mashhad, Iran; 2 Nanotechnology Research Center, Pharmaceutical Technology Institute, Mashhad University of Medical Sciences, Mashhad, Iran; 3 Department of Pediatrics, Mashhad University of Medical Sciences, Mashhad, Iran; 4 Akbar clinical research and development unit, Mashhad University of Medical Sciences, Mashhad, Iran; 5 Department of Nutrition, Faculty of Medicine, Metabolic Syndrome Research Center, Mashhad University of Medical Sciences, Mashhad, Iran

**Keywords:** Cystic fibrosis, Inflammation, Nano-curcumin, Child, Treatment outcome, Curcumin, Randomized controlled trial

## Abstract

**Background:**

Cystic fibrosis (CF) is a genetic disorder, which is caused by the CFTR protein defects. Along with CFTR dysfunction, inflammation plays a key role in the disease outcomes. Inflammation may develop due to the internal dysfunction of the CFTR protein or external factors. Curcumin affects the CFTR protein function primarily as a corrector and potentiator and secondary as an anti-inflammatory and antimicrobial agent. The present study aims to assess the impact of nano-curcumin on clinical and inflammatory markers in children with CF.

**Methods:**

This prospective, double blind control trial will be conducted at the Akbar Children’s Hospital in Mashhad, Iran. Children with CF will be enrolled based on the eligibility criteria. Placebo and curcumin with the maximum dose of 80 mg considering the body surface of the patients will be administrated for 3 months. The primary outcome is to evaluate inflammation based on serum interleukin-6, interleukin-10, and hs-CRP, stool calprotectin, and neutrophil count of nasopharyngeal swab. The secondary outcome involved clinical assessment via spirometry, anthropometrics, and quality of life. They will be assessed before and after 3 months.

**Discussion:**

Due to the multifarious effects of curcumin on CF disease, it could be proposed as a nutritional strategy in the treatment of cystic fibrosis.

**Trial registration:**

Iranian Registry of Clinical Trials IRCT20200705048018N1. Registered on July 10, 2020.

**Supplementary Information:**

The online version contains supplementary material available at 10.1186/s13063-021-05224-6.

## Administrative information

The order of the items has been modified to group similar items (see http://www.equator-network.org/reporting-guidelines/spirit-2013-statement-defining-standard-protocol-items-for-clinical-trials/).

**Table:** 

Title {1}	The Effects of Nano-curcumin as a Nutritional Strategy on Clinical and Inflammatory Factors in Children with Cystic Fibrosis: The Study Protocol for a Randomized Controlled Trial
Trial registration {2a and 2b}.	Iranian Registry of Clinical Trials, IRCT20200705048018N1, Registered on 2020-07-10
Protocol version {3}	This is the first version of protocol , 2020-07-10
Funding {4}	The vice chancellery for research of Mashhad University of Medical Sciences (MUMS)
Author details {5a}	1-Pediatrician, PhD candidate of nutrition, Department of Nutrition, Faculty of medicine, Mashhad University of Medical Sciences, Mashhad, Iran. 2-Professor of Nutrition, Department of Nutrition, Faculty of medicine, Mashhad University of Medical Sciences, Mashhad, Iran. 3-Nanotechnology Research Center, Pharmaceutical Technology Institute, Mashhad University of Medical Sciences, Mashhad, Iran. 4-Department of Pediatrics, Mashhad University of Medical Sciences, Mashhad, Iran. 5-Assistant professor of community medicine, Akbar clinical research and development unit, Mashhad University of Medical Sciences, Mashhad, Iran. 6-Department of Biological and Environmental Sciences, Faculty of life and Medical Sciences, University of Hertfordshire, Hatfield, UK. 7-Professor of Pediatric Gastroenterology, Department of Pediatrics, Mashhad University of Medical Sciences, Mashhad, Iran
Name and contact information for the trial sponsor {5b}	for research of Mashhad University of Medical Sciences (MUMS), and all study stages will be undertaken under its supervision. Tel: 009838433363 Email: ramresearch@mums.ac.ir
Role of sponsor {5c}	All study stages will be undertaken under its supervision.

## Introduction

### Background and rationale {6a}

Cystic fibrosis (CF) is a common, life-shortening, autosomal recessive, genetic disorder in Caucasian populations. The disease prognosis has improved in recent decades with the advances in the treatment procedures, and the mortality rate has decreased by 2% annually. The median age of survival in the male and female patients with CF has been reported to be 65 and 56 years, respectively [1]. Inflammation plays a critical role in CF lung pathology and disease progression. The abnormal function of cystic fibrosis transmembrane conductance regulator (CFTR) alters the chloride ion transport in the epithelial cells, hence leading to the dehydration of the airway surface liquid. This condition causes the secreted mucin to thicken and contribute to airway obstruction, thereby predisposing the patients to infection. This is considered to be the leading cause to trigger inflammation [2]. The dysregulation of the pro-inflammatory mediators secondary to the innate and adaptive immune dysfunction in CF patients is another cause of excessive inflammation. It remains unclear whether the intrinsic effects of mutant CFTR on the immune system are the major cause of inflammation or exposure to the CF airway infection and inflammatory environment could exert extrinsic effects toward this phenomenal [3, 4]. With the advent of new modified CFTR protein drugs such as ivacaftor, lumacaftor, tezacaftor, a significant impact has been observed on the treatment of patients. Ivacaftor and tezacaftor act as the potentiator through enabling the opening chloride channel, while lumacaftor acts as the corrector through increasing the trafficking of the CFTR proteins to the outer cell membrane [5, 6]. Although these drugs could reduce airway inflammation, they cannot eliminate it completely [7]. On the other hand, these drugs affect specific mutations and do not cover all patients with various mutations [7]. Therefore, anti-inflammatory treatments are an inevitable therapy for CF patients. Curcumin is the main ingredient in turmeric with a substantial impact on the reduction of inflammatory factors [8, 9]. The first study published by Egan et al. demonstrated that curcumin induced accumulation of complex-glycosylated F508 CFTR and increased cell surface density [10] Curcumin as a sarcoplasmic/endoplasmic reticulum calcium (SERCA) pump inhibitor could increase the appearance of functional DF508 CFTR on the plasma membrane cells [10]. On the other hand, curcumin suppressed the endogenous calreticulin mRNA transcription, which is a chaperone protein could bind to the mutant CTFR protein and carry this complex to the ER. so that CFTR protein can be stabilized on the cell membrane [11].

Potentiation function of curcumin has been reviewed and confirmed in a number of studies. Curcumin can react as a potentiator that repairs sub activation of CFTR protein activity and also accelerates CFTR protein activity which has been increased by other CFTR modulation in mutations such as S1251N, G551D, and F508del [12]. Curcumin can increase G551D-CFTR opening channel and its activity on its own, or when it is added to the channel that is further affected by genistein. Thus, these neutral components are suggested to have different mechanisms as potentiations of channels [13]. Similarly, curcumin stimulates channels which lack NBD2 due to deletion of G551D CFTR mutants, where effect is persistent and irreversible [14].

W1282X is another common CF mutation which reduces CFTR function by reduction of steady state levels of CFTR mRNA and also lowering the channel activity due to the defect of the second cytosolic nucleotide binding domain (NBD2) that is a surface that bind to ATP molecules [15–17]. According to several studies, curcumin stimulated W1282X-CFTR after adding a saturating dose of VX-770; thus, it increased the rate of channel opening. In addition, both curcumin and VX-770 are acting as allosteric modulators since they stimulate activity of the CFTR channel without ATP binding, but with different mechanisms [18].

According to the literature, curcumin can reduce inflammation through several mechanisms. Inflammation has a significant role in the outcome of cystic fibrosis patients. Studies have shown an extensive inflammation on pulmonary, gastrointestinal, and systemic levels in CF patients.

Mucus plugging is suitable for microorganism growth and neutrophilic inflammation as it has many nutrients. It is believed that delayed neutrophil apoptosis and long lifespan of neutrophils could increase the levels of neutrophil extracellular traps (NETs); thus, inflammation is induced [19]. Moreover, differentiation of monocyte in to macrophage and division into M1 or M2 phenotype are unsuccessful in CF patients, and hence, it does not produce IL-13Rα1 on the surface. This impaired process contributes to excessive inflammation in CF lung disease [20]. Last but not least, the highest number of Th17 cells is present in the CF lung. Th17 cells produce both IL-8 and IL-17 and promote neutrophil accumulation. These T cells are activated by IL-6, IL-23, and TGF-β and suppressed by active T regulatory-lymphocytes (Treg). Otherwise, a CFTR abnormality or an unknown imbalance between Th17 cells and Treg can induce inflammation by neutrophil recruitment [2].

Anti-bacterial effect of curcumin has also been considered in several studies. Toll-like receptor-2(TLR2) is a receptor of pathogenic bacteria like *Staphylococcus aureus* peptidoglycan (PGN), is found in epithelial cell and has a key role in recognizing the infected pathogen, increases inflammatory cytokines as a result, and is upregulated in CF patients. Cystic fibrosis patients are prone to infection since absence of CFTR protein causes demethylation of DNA at the specific CpG sites which overlaps a minimal region to maintain activity of TLR2 promoter [21]. Curcumin can degrade specificity protein 1 (SP1) via oxidative and proteasome degradation pathways. This factor is essential for upregulation of TLR2 expression. Consequently, curcumin decreases basal expression of TLR2 [22]. Finally, curcumin has been proved to have a significant effect on suppressing the growth of *P. aeruginosa* on biofilm optical densities, although the MIC (minimum bacterial concentration) which is needed for growth suppression is higher than other usual *P. aeruginosa* treatments such as imipenem or tobramycin [23].

The above evidence suggests that curcumin has potential effects on CF patients.

It must be considered that curcumin is soluble in water and has a short half-life and low bioavailability. Various products have been obtained for the improvement of bioavailability through increasing the absorption rate from the gastrointestinal tract and reducing the liver metabolism [24–26].

The present study aimed to evaluate the effects of nano-curcumin on CF patients in order to achieve the intrinsic and extrinsic effects of curcumin on the inflammatory process in these patients.

### Objectives {7}

The present study aimed to evaluate the effects of nano-curcumin on CF patients in order to achieve the intrinsic and extrinsic effects of curcumin on the inflammatory process in these patients.

### Trial design {8}

This is a prospective, single center, double-blind, parallel, randomized placebo-controlled clinical trial. The study protocol was written following the standard Protocol Items Recommendation for Interventional Trials (SPIRIT) Checklist (Table 1).

**Table 1:**
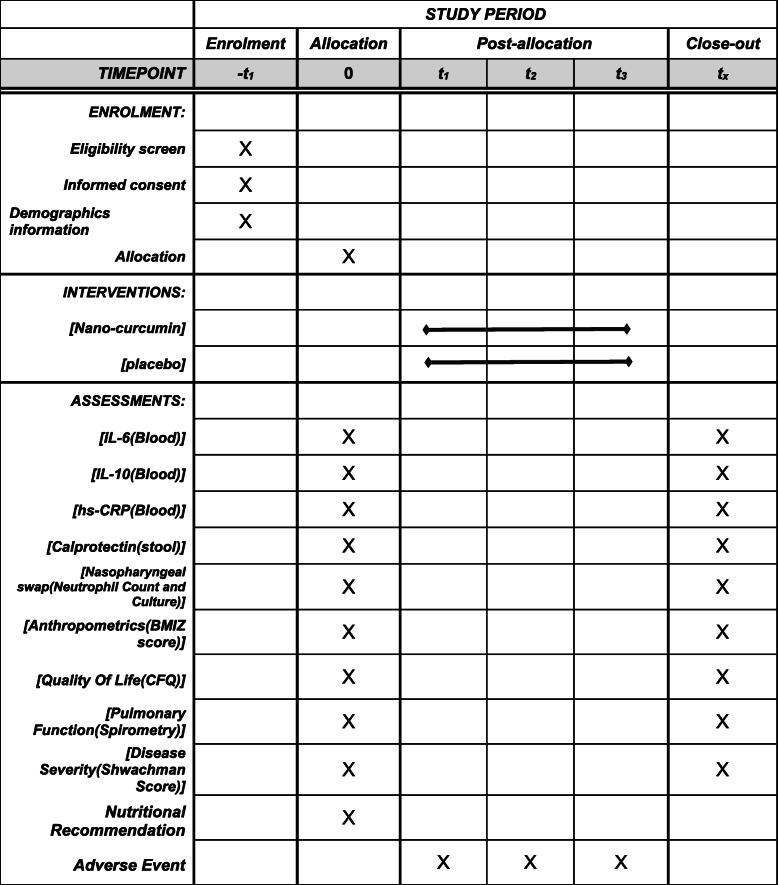
Schedule of enrolment, intervention, and assessment based in the Standard Protocol Items Recommendations for Interventional Trials (SPIRIT) guidelines

## Methods: participants, interventions, and outcomes

### Study setting {9}

The study population will consist of the patients with known CF referring to the Cystic Fibrosis Clinic at Akbar Children’s Hospital, Mashhad, Iran.

### Eligibility criteria {10}

#### Inclusion criteria

The inclusion criteria of the study are as follows: (1) one or more typical phenotypic features of CF and a minimum of an elevated sweat chloride concentration on two/more occasions or two mutations known to cause CF on separate alleles; (2) age of 5–18 years; (3) pulmonary and gastrointestinal involvement; (4) ability to perform spirometry maneuvers and the minimum FEV1 of ≥ 30% compared to the same age, gender, and height in the normal population; (5) the percentage of oxygen saturation based on pulse the oximetry of ≥ 90% at room temperature; and (6) informed consent for participation.

#### Exclusion criteria

The exclusion criteria of the study are (1) cardiovascular, hepatic, and renal failure; (2) celiac disease and rheumatoid arthritis; (3) acute pulmonary exacerbation requiring hospitalization within the past 4 weeks; and (4) acute respiratory tract infection.

#### Who will take informed consent? {26a}

Initially, the objectives of the trial will be clarified by the first researcher to the children’s parents and legal guardians, and written informed consent will be obtained from them at the time of enrolment.

#### Additional consent provisions for collection and use of participant data and biological specimens {26b}

In the consent form, the participants will be asked whether they agree upon using their data in case they withdraw from the trial. In addition, their permission will be obtained by the research team to share relevant data with the academic experts selected from the universities partaking in the research or other regulatory authorities. Notably, this trial does not involve the collection of biological specimens for storage.

### Interventions

#### Explanation for the choice of comparators {6b}

The participants in the placebo/control group will receive standard CF treatments, and curcumin will be used as add-on therapy; this is the reason for considering placebo as the control group in our study.

#### Intervention description {11a}

Nano-curcumin (Exir Nano Sina Drug Company, Iran) is prepared as nano micelle in the form of 70 mg of drops in 1 cc, and the placebo with the same color, taste, and odor.

To adjust the drug dose for different ages and considering that the maximum acceptable dose based with the most significant impact and minimum side effects was 80 mg, the required amount will be obtained for each subject based on the ratio of the body surface area of the patients. The curcumin and placebo glasses have been labeled A and B by Exir Nano Sina company, respectively, and made available to the patients through a double-blind design. Curcumin or placebo will be administered orally alone or with sweetened water once daily at bedtime and at a fixed dose for 3 months.

#### Criteria for discontinuing or modifying allocated interventions {11b}

Although no special side effect has been reported until now, with any intervention-related side effects, we will stop the intervention and report it to the Ethics committee of Mashhad University of Medical Sciences (MUMS) for decision making. If the participants request to discontinue treatment or report the deterioration of their condition due to CF, we will stop the intervention.

#### Strategies to improve adherence to interventions {11c}

The strategies adopted to improve the adherence of the patients to the intervention include taking the medicine once a day versus several times, using an oral drop versus a soft gel for easier consumption by children and using the medications and prescribed agents before bedtime so as to minimize interference with other common CF medications. In addition, the individualized training of the patients and their children was performed on the benefits and necessity of the accurate and complete use of the intervention for an optimal impact. The lack of adherence to the drug occurs in cases where less than 70% of the prescribed dose is used, which will be assessed during monthly follow-up sessions.

#### Relevant concomitant care permitted or prohibited during the trial {11d}

Concomitant intervention will be:

1. Pancreatic enzyme replacement therapy (PERT): All CF patients with pancreatic deficiency need PERT for enzyme replacement. In this regard, we enrolled CF patients with gastrointestinal involvement; all of our patients use Creon as a usual treatment in which doses are adjusted to the level of dietary fat.
2. Antibiotic: Tobramycin inhalation is an antibiotic which is used for pseudomonas infection.
3. Anti-inflammatory: Low-dose azithromycin treatment frequently prescribed chronically that has an anti-inflammatory effect in CF patients 6 years and older. We use it in our patients.
4. Anti-acid: Suggested for patients who fail to respond to maximal dose of PERT and in patients with reflux disease.
5. Inhaled hypertonic saline: Nebulized hypertonic (7%) saline is recommended twice daily to all patients 6 years and older.
6. Inhaled beta2 agonist: Administrated in CF patients with moderate to severe lung disease twice a week.
7. Fat soluble vitamins: For all CF patients with pancreatic deficiency. Notably, the administration of the nano-curcumin drop or nano-curcumin placebo drop will not require any changes in the routine treatment of CF, and the care procedure and medication will continue for both trial arms.

#### Provisions for post-trial care {30}

There is no anticipated harm and compensation for participation in this trial.

#### Outcomes {12}

The primary outcomes of the current trial are the compared changes in the blood vessel inflammation, nasopharyngeal swab, and fecal samples at baseline with 3 months after the intervention: systemic inflammation by assessing IL-6 as an inflammatory agent, IL-10 as an anti-inflammatory agent, and hs-CRP level in the blood samples, pulmonary inflammation with the neutrophil count, bacterial/viral culture on the nasopharyngeal swab, and gastrointestinal inflammation with the calprotectin level in the fecal samples.

The secondary outcomes are changes in clinical assessment of the pulmonary symptoms via spirometry, anthropometric assessment stand on the BMI Z score, and evaluation of the quality of life using the CFQ from the baseline to 3 months of the intervention.

#### Participant timeline {13}

Nutritional recommendations will be provided to all the patients and/or their parents and legal guardians stand on the CF requirements and adjustable CREON dosing to the level of dietary fat. In addition, the patients and their parents completed the CFQ depend on age.

Anthropometric measurements (weight and height) will be performed using a digital scale (model: SECA). Body weight will be measured without shoes, and height will be measured in the standing position without shoes with the heels stuck to the wall and the head looking frontwards with the accuracy of 0.5 cm.

The fecal examination will be performed before and after the intervention. Before intervention, fecal sample will be evaluated for bacterial over growth and parasite specially giardia by checking stool PH and trophozoites or cysts of giardia. Any positive result will be treated with antibiotics before stating the trials.

Blood sampling will be performed before and after the intervention. Approximately 5 mL of blood will be collected, immediately centrifuged, with the serum separated from the sediment, and preserved at the minimum of temperature of − 20 °C.

The primary and secondary consequences of the treatment will be investigated before and 3 months after the treatment. The clinical evaluation and follow-up of medication use and side effects will also be carried out via phone call and paying monthly visit to the clinic, and the findings will privately be presented to the patients (Table 2).

**Table 2:** Laboratory measurements, equipment, and normal range

Test	Test equipment	Company	Quality control	Normal range
**Hs-CRP**	Biochemical auto analyzer	Pars azmoon	Intra-assay CV: 2.2% Inter-assay CV: 1.1% Linearity (*R*^2^): 0.99 Detection limit: 0.1 mg/lit	Children: up 2.8 mg/L Adult: up to 5 mg/L
**IL-6**	ELISA kit	Demeditec	Intra assay CV: 4.2% Inter assay CV: 4.9% Accuracy: 99% Specificity: 94% Detection limit: 2 pg/ml	Up to 1.8 pg/ml
**IL-10**	ELISA kit	pars gene (Invitrogen)	Intra assay CV < 3% Inter assay CV < 8% Detection limit:2 pg/ml	1.2–7.8 pg/ml
**Calprotectin**	ELISA kit	EUROIMMUN	Intra assay CV: 3.4% Inter assay CV: 6.4% Linearity(*R*^2^): 0.99 Sensitivity: 94.1% Specificity: 95.5% Detection limit:1.9 μg/g	50–120 μg/g: borderline > 120 μg/g: positive
**Nasopharyngeal swab**	URT-S&C (gram-positive, gram negative, pseudomonas)	Variable		Culture: negative WBC count: up to 2–3

#### Sample size {14}

Due to the lack of clinical trials on the inflammatory effect of nano-curcumin in children with cystic fibrosis, we used HSCRP indices as an effect size. Therefore, the mean ± SD of hs-CRP indices in the curcumin and placebo groups were 5.9 ± 2.57 and 3.6 ± 1.58 respectively [27]. G- power analyzer was applied for calculating sample size between two independent populations with a confidence interval of 95% and power of 80%. By considering 10% dropout and using Deff (deign effect) for stratified sample, a total of 30 samples for each group was calculated. In total, 60 eligible patients with CF are needed to be included for the randomization aspect of this research.

#### Recruitment {15}

Our patients will be selected from the cases referring to a specialized CF clinic, which covers all the CF patients in our regional state. These patients are also supported by the Local CF Association. The initial data are available on the CF registry. As a result, access to the patients and their follow-up will be easier.

### Assignment of interventions: allocation

#### Sequence generation {16a}

**Fig. 1:**
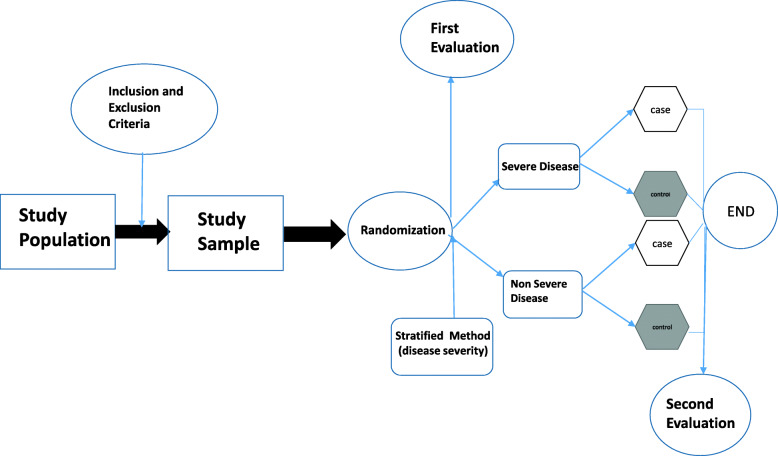
Schematic of study design. First and second evaluations consist of the following: serum interleukin-6 (IL-6), IL-10, and high sensitivity. C-reactive protein (hs-CRP), fecal calprotectin, weight and body mass index (BMI), and score of quality of life of the cystic fibrosis questionnaire (CFQ)

The patients meeting the inclusion criteria will be enrolled in the study. Initially, stratified randomization will be used to divide the participants into two groups based on disease severity (severe/mild-to-moderate) using spirometry with 40% FEV1 cutoff range. Following that, random sampling will be applied to select the patients from each group to be administered with the nano-curcumin drop or nano-curcumin placebo drop using a random number table (Fig. 1).

#### Concealment mechanism {16b}

The random number table is used to select the patients from each group following stratified randomization based on disease severity. In addition, a random sequence is printed and placed inside envelopes by an individual other than the trial researchers. The envelopes will be randomly selected by the patients, so that they would be assigned to the intervention or control groups based on the contents of the selected envelope. Curcumin and placebo labeling has also been provided by Exir Nano Sina Company.

#### Implementation {16c}

Generation of the allocation sequence, enrollment of participants, and assignment of participants to interventions will be done by the main researcher of the trial.

### Assignment of interventions: blinding

#### Who will be blinded {17a}

Curcumin and placebo glasses have been labeled A and B by Exir Nano Sina Company, respectively, and are made available to the patients through the double-blind design of the trial. Notably, the participants and researchers are unaware of the groups assigned to the patients, while the data analysts are not blinded to the trial.

#### Procedure for unblinding if needed {17b}

Although we do not anticipate any requirement for non-blinding, the trial manager, data coordinator, implementation support facilitators, participants, and researchers will have access to group allocations, and possible non-blinding will be reported.

### Data collection and management

#### Plans for assessment and collection of outcomes {18a}

As the primary outcome of the present study, blood vessel inflammation will be assessed in the sample using the ELISA assay (IL-6, IL-10, and hs-CRP), stool inflammation will be determined by measuring the calprotectin level using the ELISA assay, and pulmonary inflammation will be evaluated using the smear and culture (gram-positive, gram-negative, pseudomonas) of the nasopharyngeal soap. All the tests will be performed at the Buali Research Institute by expert personnel. Table 2 shows the performance specifications of the applied methods.

For the quality assurance of the employed laboratory methods, the performance specifications will be verified before using the kits for the analysis of the patient samples. All the tests will be performed in duplicate, and the standard curve will be prepared using the serial dilutions of the standard samples. The obtained results will be calculated from the standard curve after adjustment for the blank.

The secondary outcome of the present study was anthropometric assessment, which will be performed in a standard position using SECA instruments for height and weight. In addition, the WHO growth standard charts will be used for the evaluation of the BMI *z*-score.

In the current trial, we will apply the CFQ-R to assess the effects of nano-curcumin on the quality of life of the children with CF. The CFQ was first developed in France and consists of three appropriate versions for children aged 6–13 years (CFQ-child), as well as the parents of children with CF aged 6–13 years (CFQ-parent) and adolescents and adults with CF aged 14 years (CFQ-teen/adult) [28]. After the psychometric assessment of the original version and slight modifications, the final version of the CFQ-R was developed.

Spirometry will be performed using a spirometer (CHESTGRAPH HI-105), with the patients in a stable, standing position and their neck completely straight. To increase accuracy and reliability, the test will be repeated at least twice. If FVC and FEV1 are more than 150 ml different from the first time, the test will be repeated; the maximum repetition will be eight times.

#### Strategies to promote participant retention and complete follow-up {18b}

Due to the fact that our patients are selected from the pediatric population, their parents will be asked to provide written feedback on the observed outcomes of the evaluations in their children in addition to the regular follow-up via phone or free-of-charge visits.

#### Data management {19}

In the current trial, the database will be generated from the questionnaire of clinical data and laboratory assay results in the Excel software and SPSS. As the trial researchers, selected and trained nurses and assistants will collect the data at baseline and during the follow-up and record the obtained data in the paper questionnaires, as well as the designed Excel datasheet. At the next stage, the data will be analyzed in the SPSS. To ensure security, the login passwords are allowed only for the trial researchers. The database is prepared to only accept the variables that are consistent with the previous entries, and it could also be altered in the case of randomly missing values. Twice a month, 10% of the electronic data will be randomly compared with the paper questionnaires, and discrepancies will lead to a 50% double-checking of the electronic data.

#### Confidentiality {27}

Data of the participants will be stored at the security site. The laboratory specimens, collected data, and reports will be identified by a coded ID number. Data collected during the course of the research will remain strictly confidential and only accessed by the research team. The corresponding author will also have access rights to the dataset. In addition, the anonymous data will be shared with other researchers to enable international prospective meta-analyses.

#### Plans for collection, laboratory evaluation, and storage of biological specimens for genetic or molecular analysis in this trial/future use {33}

This trial does not involve the collection of biological specimens for storage.

#### Statistical methods for primary and secondary outcomes {20a}

Data analysis will be performed in SPSS version 16, and data distribution will be evaluated using the Kolmogorov-Smirnov test. Data are expressed as frequencies for the qualitative variables, mean and standard deviation for the normally distributed continuous variables, and median and interquartile range for the other variables. In addition, intergroup differences will be evaluated using paired t-test for the normally distributed continuous variables and Wilcoxon rank-sum test for the other variables. Intragroup differences will be assessed using independent *t* test for the normally distributed continuous variables and Mann-Whitney *U* test for the other variables. The mean changes in the secondary outcomes will also be adjusted for the baseline data and disease severity using the analysis of covariance (ANCOVA). In all the statistical analyses, the *P* value of ≤ 0.05 will be considered significant.

#### Interim analyses {21b}

Interim analysis is not considered. In case of frequent side effects (more than previous reports), we will stop the intervention and present the results to the Ethics Committee of Mashhad University of Medical Sciences (MUMS) for further decision

#### Methods for additional analyses (e.g., subgroup analyses) {20b}

The baseline data and disease severity will be controlled using ANCOVA or binary logistic regression.

#### Analytical method to handle protocol non-adherence and any statistical methods to handle missing data {20c}

In order to incorporate the outcome data of the participants into data analysis regardless of the protocol adherence, all the analyses will be performed in accordance with the intention-to-treat principle. The final observation by the forward method will also be used to impute the missing data for the intention-to-treat analysis.

#### Plans to give access to the full protocol, participant level-data and statistical code {31c}

We will attempt to release the full study protocol and results as soon as possible, regardless of the magnitude or direction of effect. The anonymized data set and statistical code may be available from the corresponding author (Email: kianifar HR@mums.ac.ir) on reasonable request.

### Oversight and monitoring

#### Composition of the coordinating center and trial steering committee {5d}

The ethical committee and vice chancellery of Mashhad University of Medical Sciences supervise all the study stages. It is an academic committee and has no competing interest

#### Composition of the data monitoring committee, its role and reporting structure {21a}

The Data Monitoring Committee was not involved in the current trial considering the low-risk intervention.

#### Adverse event reporting and harms {22}

No severe adverse events are anticipated although potential minor events may occur, which will be reported to the Ethics Committee of Mashhad University of Medical Sciences (MUMS) for decision-making.

#### Frequency and plans for auditing trial conduct {23}

The Ethics Committee of MUMS will be fully aware of the trials from the first draft of the protocol to the end of the research process and will monitor the accuracy of the trials at least twice.

#### Plans for communicating important protocol amendments to relevant parties (e.g., trial participants, ethical committees) {25}

Any modification to protocol which may impact on the conduct of the study will be approved by the ethical committee of MUMS prior to implementation.

#### Dissemination plans {31a}

The process of the approval and submission of the reports for dissemination via journal publication will last about 4–6 months, and the results will be disseminated regardless of the magnitude or direction of their effects. Furthermore, all the conditions related to publishing the results of the trial pertain to the corresponding author, and there are no publication restrictions imposed by the sponsor.

## Discussion

Curcumin [1,7-bis (4-hydroxy-3-methoxyphenyl)-1,6-heptadiene-3,5-dione] is a polyphenolic compound isolated from the rhizomes of *Curcuma longa* (turmeric) [29].

Secondary metabolites of this component are phenolic acids, flavonoids, alkaloids, terpenoids, tannins, and saponins, all of which are known to have biological effects. The antibacterial effect of curcumin has been exhibited since 1949 [30], and several studies have revealed its anti-inflammatory, anticancer, antioxidant, wound-healing, and antiviral effects which are believed to have therapeutic effects in many human-related diseases [31]. Curcumin is suggested as a line of treatment for cystic fibrosis disease. Since CF is the most common life shortening disease, early initiation of treatments, including anti-inflammatory agent, is very important in the final prognosis of the disease. It appears that cystic fibrosis children are the best candidates for using this treatment.

**Fig. 2:**
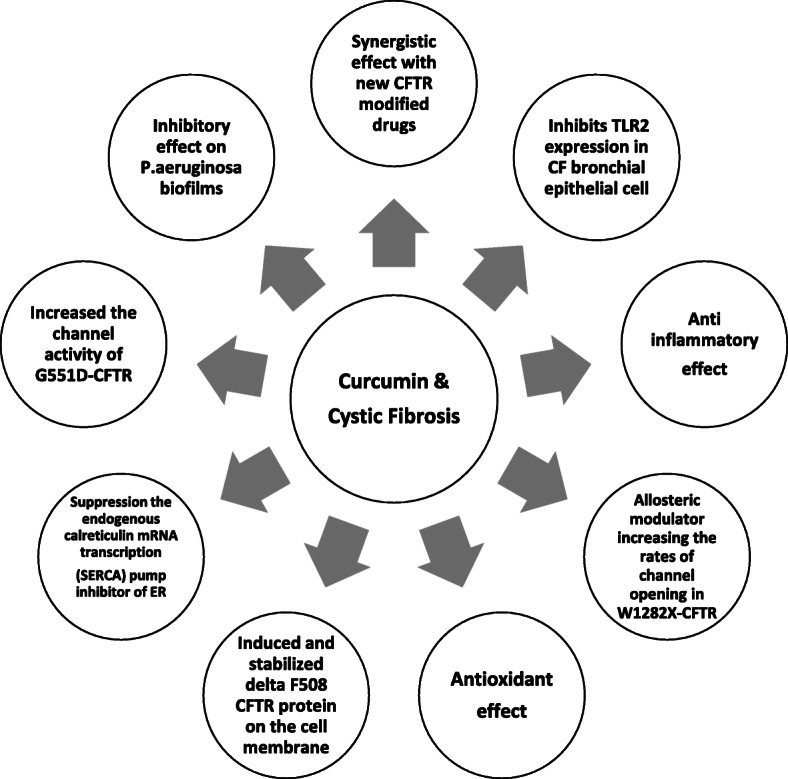
Diagram effects of curcumin in cystic fibrosis disease as a CFTR modulator, anti-inflammatory, antioxidant, and anti-microbial

According to previous research, it is shown that cystic fibrosis transmembrane conductance regulator (CFTR) is a cAMP-activated Cl ion channel in the apical membrane of epithelial cells [32], which is a well-studied ion channel target for curcumin (Fig. 2).

Since the reduction of inflammation plays a key role in the conditions of patients with CF and considering the primary effects of curcumin on the CFTR protein, extensive investigations are required regarding the applications of this natural compound in these patients. Recently, the nano-curcumin formulation has been extensively studied considering its impact on the increased bioavailability of curcumin. This formulation has been reported to have a great effect on the reduction of inflammatory processes.

The main limitation of the current study is the lack of assessment of CFTR protein function by nasal epithelial biopsy and cellular evaluation. However, this method is invasive and cannot be performed easily on children. To the best of our knowledge, this is the first study which will evaluate the use of curcumin as a specific and supportive nutritional agent in children with CF.

## Supplementary Information

**Additional file 1.**

## Abbreviations

IL: Interleukin
hs-CRP: High sensitive C-reactive protein
BMI: Body mass index
CFQ: Cystic Fibrosis Questionnaires
CFTR: Cystic fibrosis transmembrane conductance regulator
RCT: Randomize control trial
FEV1: Forced expiratory volume 1
PERT: Pancreatic enzyme replacement therapy
Camp: Cyclic adenosine monophosphate
SERCA: Sarco/endoplasmic reticulum Ca ATPase
ER: Endoplasmic reticulum
ROS: Reactive oxygen species
TGF: Transforming growth factor
M: Mitosis
TLR: Toll-like receptor
MAPK: Mitogen-activated protein kinase
RNS: Reactive nitrogen species
NBDs: Nucleotide-binding domains
MIC: Minimum inhibitory concentration
NETs: Neutrophil extracellular traps

## References

[1] Keogh RH, Szczesniak R, Taylor-Robinson D, Bilton D (2018). Up-to-date and projected estimates of survival for people with cystic fibrosis using baseline characteristics: a longitudinal study using UK patient registry data. J Cystic Fibrosis.

[2] Roesch EA, Nichols DP, Chmiel JF (2018). Inflammation in cystic fibrosis: an update. Pediatr Pulmonol.

[3] Cohen TS, Prince A (2012). Cystic fibrosis: a mucosal immunodeficiency syndrome. Nat Med.

[4] Lubamba BA, Jones LC, O'Neal WK, Boucher RC, Ribeiro CM (2015). X-box-binding protein 1 and innate immune responses of human cystic fibrosis alveolar macrophages. Am J Respir Crit Care Med.

[5] Connett GJ (2019). Lumacaftor-ivacaftor in the treatment of cystic fibrosis: design, development and place in therapy. Drug Des Devel Ther.

[6] Sala MA, Jain M (2018). Tezacaftor for the treatment of cystic fibrosis. Expert Rev Respir Med.

[7] Jarosz-Griffiths HH, Scambler T, Wong CH, Lara-Reyna S, Holbrook J, Martinon F, et al. Different CFTR modulator combinations downregulate inflammation differently in cystic fibrosis. eLife. 2020;9. 10.7554/eLife.54556. 10.7554/eLife.54556 PMC7062465 32118580

[8] Dey I, Shah K, Bradbury NA. Natural compounds as therapeutic agents in the treatment cystic fibrosis. J Genetic Syndr Gene Ther. 2016;7(1). 10.4172/2157-7412.1000284. 10.4172/2157-7412.1000284 PMC4828912 27081574

[9] Lelli D, Sahebkar A, Johnston TP, Pedone C (2017). Curcumin use in pulmonary diseases: state of the art and future perspectives. Pharmacol Res.

[10] Egan ME, Pearson M, Weiner SA, Rajendran V, Rubin D, Glöckner-Pagel J, Canny S, du K, Lukacs GL, Caplan MJ (2004). Curcumin, a major constituent of turmeric, corrects cystic fibrosis defects. Science..

[11] Harada K, Okiyoneda T, Hashimoto Y, Oyokawa K, Nakamura K, Suico MA, Shuto T, Kai H (2007). Curcumin enhances cystic fibrosis transmembrane regulator expression by down-regulating calreticulin. Biochem Biophys Res Commun.

[12] Dekkers JF, Van Mourik P, Vonk AM, Kruisselbrink E, Berkers G, de Winter-de Groot KM (2016). Potentiator synergy in rectal organoids carrying S1251N, G551D, or F508del CFTR mutations. J Cyst Fibros.

[13] Yu YC, Miki H, Nakamura Y, Hanyuda A, Matsuzaki Y, Abe Y, Yasui M, Tanaka K, Hwang TC, Bompadre SG, Sohma Y (2011). Curcumin and genistein additively potentiate G551D-CFTR. J Cystic Fibrosis.

[14] Wang W, Bernard K, Li G, Kirk KL (2007). Curcumin opens cystic fibrosis transmembrane conductance regulator channels by a novel mechanism that requires neither ATP binding nor dimerization of the nucleotide-binding domains. J Biol Chem.

[15] Linde L, Boelz S, Nissim-Rafinia M, Oren YS, Wilschanski M, Yaacov Y, Virgilis D, Neu-Yilik G, Kulozik AE, Kerem E, Kerem B (2007). Nonsense-mediated mRNA decay affects nonsense transcript levels and governs response of cystic fibrosis patients to gentamicin. J Clin Invest.

[16] Berger AL, Ikuma M, Welsh MJ (2005). Normal gating of CFTR requires ATP binding to both nucleotide-binding domains and hydrolysis at the second nucleotide-binding domain. Proc Natl Acad Sci U S A.

[17] Sikora-Polaczek M, Bielak-Zmijewska A, Sikora E (2011). Molecular and cellular mechanisms of curcumin action-beneficial effect on organism. Postepy Bioch.

[18] Wang W, Hong JS, Rab A, Sorscher EJ, Kirk KL (2016). Robust stimulation of W1282X-CFTR channel activity by a combination of allosteric modulators. PLoS One.

[19] Gray RD, Hardisty G, Regan KH, Smith M, Robb CT, Duffin R, Mackellar A, Felton JM, Paemka L, McCullagh BN, Lucas CD, Dorward DA, McKone EF, Cooke G, Donnelly SC, Singh PK, Stoltz DA, Haslett C, McCray PB, Whyte MKB, Rossi AG, Davidson DJ (2018). Delayed neutrophil apoptosis enhances NET formation in cystic fibrosis. Thorax..

[20] Tarique AA, Sly PD, Holt PG, Bosco A, Ware RS, Logan J, Bell SC, Wainwright CE, Fantino E (2017). CFTR-dependent defect in alternatively-activated macrophages in cystic fibrosis. J Cyst Fibros.

[21] Shuto T (2013). Regulation of expression, function, and inflammatory responses of innate immune receptor Toll-like receptor-2 (TLR2) during inflammatory responses against infection. Yakugaku Zasshi.

[22] Chaudhary N, Ueno-Shuto K, Ono T, Ohira Y, Watanabe K, Nasu A, et al. Curcumin down-regulates toll-like receptor-2 gene expression and function in human cystic fibrosis bronchial epithelial cells. Biol Pharm Bull. 2019;42(3):489–95. 10.1248/bpb.b18-00928. 10.1248/bpb.b18-00928 30626802

[23] Karaman M, Fırıncı F, Arıkan Ayyıldız Z, Bahar IH (2013). Effects of imipenem, tobramycin and curcumin on biofilm formation of Pseudomonas aeruginosa strains. Mikrobiyoloji bulteni.

[24] Cartiera MS, Ferreira EC, Caputo C, Egan ME, Caplan MJ, Saltzman WM (2010). Partial correction of cystic fibrosis defects with PLGA nanoparticles encapsulating curcumin. Mol Pharm.

[25] Yallapu MM, Nagesh PK, Jaggi M, Chauhan SC (2015). Therapeutic applications of curcumin nanoformulations. AAPS J.

[26] Flora G, Gupta D, Tiwari A (2013). Nanocurcumin: a promising therapeutic advancement over native curcumin. Crit Rev Ther Drug Carrier Syst.

[27] Jazayeri-Tehrani SA, Rezayat SM, Mansouri S, Qorbani M, Alavian SM, Daneshi-Maskooni M (2019). Nano-curcumin improves glucose indices, lipids, inflammation, and Nesfatin in overweight and obese patients with non-alcoholic fatty liver disease (NAFLD): a double-blind randomized placebo-controlled clinical trial. Nutr Metab (Lond).

[28] Henry B, Aussage P, Grosskopf C, Goehrs J-M (2003). Development of the Cystic Fibrosis Questionnaire (CFQ) for assessing quality of life in pediatric and adult patients. Qual Life Res.

[29] Hewlings SJ, Kalman DS (2017). Curcumin: a review of its’ effects on human health. Foods..

[30] Adamczak A, Ożarowski M, Karpiński TM. Curcumin, a natural antimicrobial agent with strain-specific activity. Pharmaceuticals (Basel, Switzerland). 2020;13(7). 10.3390/ph13070153. 10.3390/ph13070153 PMC7408453 32708619

[31] Mantzorou M, Pavlidou E, Vasios G, Tsagalioti E, Giaginis C (2018). Effects of curcumin consumption on human chronic diseases: a narrative review of the most recent clinical data. Phytother Res.

[32] Li C, Naren AP (2010). CFTR chloride channel in the apical compartments: spatiotemporal coupling to its interacting partners. Integr Biol (Camb).

