# Supplementary material for: The effects of nano-curcumin as a nutritional strategy on clinical and inflammatory factors in children with cystic fibrosis: the study protocol for a randomized controlled trial
Source: Trials. 2021 Apr 20;22:292. doi: 10.1186/s13063-021-05224-6 (PMC8056493; doi:10.1186/s13063-021-05224-6)
Supplement: Supplementary file 1 — Additional file 1. [file 13063_2021_5224_MOESM1_ESM.pdf]

عنوان طرح پژوهشی:	نام مجری یا مجریان طرح:

**معرفی پژوهش**	
**مزایا**	
**خطرات**	
**جبران خطرات**	
**نمونه گیری،دارودرمانی یا سایر خدمات(ذکرشود)**	
**محرمانه بودن**	
**پاسخگویی به پرسشها**	
**حق انصراف درخروج ازمطالعه**	شرکت من درمطالعه کاملاً اختیاری است وآزاد خواهم بود که از شرکت درمطالعه امتناع نموده یا هرزمان مایل بودم بدون آنکه تغییری درنحوه رفتار پزشک درمانگر یا نحوه درمان ومراقبت ازبیماری اینجانب ایجاد شود ازپژوهش مذکور خارج شوم.

((رضایت))

اینجانب   با آگاهی کامل ازموارد فوق رضـــایت میدهم که به عنوان یک فرد مورد مطالعه درپژوهش به سرپرستی   شرکت نمایم .

کلیه اطلاعاتی که از من گرفته میشـود ونیز نام من محرمانه باقی خواهد ماند ونتایج تحقیقات به صـورت کلی ودرقالب اطلاعات گروه مورد مطالعه منتشر میگردد ونتایج فردی درصورت نیاز بدون ذکر نام ومشخصات فردی عرضه خواهد گردید وهمچنین برائت پزشک یا پزشکان این طرح را ازکلیه اقدامات مذکور دربرگه اطلاعاتی درصورت عدم تقصیر درارائه اقدامات اعلام میدارم.

این موافقت مانع ازاقدامات قانونی اینجانب درمقابل (نام واحد ذکر گردد) درصـورتی که عملی خلاف وغیر انسـانی انجام شـود نخواهد بود.

امضاء واثر انگشت فرد مورد پژوهش **نام و نام خانوادگی وامضاء شاهد**

امضاء پژوهشگر

آدرس و شماره تماس بیمار حتما ذکر گردد:
